# Facile Synthesis of Pd@PtM (*M* = Rh, Ni, Pd, Cu) Multimetallic Nanorings as Efficient Catalysts for Ethanol Oxidation Reaction

**DOI:** 10.3389/fchem.2021.683450

**Published:** 2021-05-19

**Authors:** Xingqiao Wu, Xiao Li, Yucong Yan, Sai Luo, Jingbo Huang, Junjie Li, Deren Yang, Hui Zhang

**Affiliations:** ^1^State Key Laboratory of Silicon Materials, Cyrus Tang Center for Sensor Materials and Applications, School of Materials Science and Engineering, Zhejiang University, Hangzhou, China; ^2^BTR New Material Group CO., LTD., Shenzhen, China; ^3^Hangzhou Innovation Center, Institute of Advanced Semiconductors, Zhejiang University, Hangzhou, China

**Keywords:** multimetallic nanocrystals, epitaxial growth, interfacial catalytic reactions, nanorings, electrocatalysis

## Abstract

Pt-based multimetallic nanorings with a hollow structure are attractive as advanced catalysts due to their fantastic structure feature. However, the general method for the synthesis of such unique nanostructures is still lack. Here we report the synthesis of Pd@PtM (*M* = Rh, Ni, Pd, Cu) multimetallic nanorings by selective epitaxial growth of Pt alloyed shells on the periphery of Pd nanoplates in combination with oxidative etching of partial Pd in the interior. *In situ* generation of CO and benzoic acid arising from interfacial catalytic reactions between Pd nanoplates and benzaldehyde are critical to achieve high-quality Pt-based multimetallic nanorings. Specifically, the *in-situ* generated CO promotes the formation of Pt alloyed shells and their epitaxial growth on Pd nanoplates. In addition, the as-formed benzoic acid and residual oxygen are responsible for selective oxidative etching of partial Pd in the interior. When evaluated as electrocatalysts, the Pd@PtRh nanorings exhibit remarkably enhanced activity and stability for ethanol oxidation reaction (EOR) compared to the Pd@PtRh nanoplates and commercial Pt/C due to their hollow nanostructures.

## Introducion

Direct ethanol fuel cells (DEFCs) are an attractive green power source for portable electronic devices since ethanol is a non-toxic liquid with high energy density and easy to be stored and transported (Antolini, 2007; Brouzgou et al., 2012). So far, platinum (Pt) have proved to be the most active monometallic catalyst for the ethanol oxidation reaction (EOR). Unfortunately, Pt is highly expensive and extremely scare in earth, severely limiting its extensive use in DEFCs. In addition, Pt is vulnerable to some intermediates and reaction byproducts (e.g., CO) during the EOR process, which will dramatically decrease its catalytic activity (Iwasita and Pastor, 1994; De Souza et al., 2002; Farsadrooh et al., 2018). These limitations push us to design advanced Pt-based catalysts with excellent catalytic properties and optimal utilization efficiency of Pt for EOR. One of the most promising strategies is to boost the intrinsic catalytic ability of noble-metal atoms by optimizing the composition, surface structure, and strain distribution (Kua and Goddard, 1999; Rossmeisl et al., 2012). Another method is to improve the specific surface area by lowering the dimension of materials (Zhang et al., 2019).

Recently, hollow nanostructures consisting of noble-metals had received increasing interest as promising electrocatalysts due to their higher specific surface area than that of solid one (Fan et al., 2015; Prieto et al., 2016; Chen et al., 2018; Li et al., 2018). In general, noble-metal hollow nanostructures were synthesized by sacrifice template assisted approaches, which can be classified into two categories depending on removal of template simultaneously with or after the formation of the outer shells (Xia et al., 2013, 2017; Chee et al., 2017). Last decade has witnessed the successful synthesis of noble-metal hollow nanostructures with various shapes through galvanic replacement and Kirkendall effect (Sun et al., 2002, 2004; Yang et al., 2016; Huang et al., 2020). Another effective approach involves the synthesis of core-shell nanocrystals first, and then removal of the core by chemical or electrochemical etching. For instance, Xia and co-workers designed a kinetically controlled method that epitaxially deposit Pt shells on Pd nanocrystals with different shapes, and the Pt nanocages were generated by removing Pd cores via chemical etching with Fe^3+^ ions in an acidic environment (Zhang et al., 2015; Wang et al., 2016a). Such nanocages exhibited the substantially enhanced catalytic activity for oxygen reduction reaction relative to Pt/C. As a hollow nanostructure, metallic nanorings have received particular interest in electrocatalysis due to their outstanding features including the effective utilization of metal atom of surface and convenient accessibility for the reactants (Fan et al., 2012; Liu et al., 2013; Wang et al., 2016b). To this end, Xie and co-workers have reported one-pot approach for the synthesis of ultrathin PdPtCu trimetallic nanosheets and nanorings, with the nanorings being more active for EOR due to their larger proportion of low-coordinated sites (Wang et al., 2020). Although significant advances have been made in the synthesis of metallic nanorings, it still lacks a universal method to generate Pt-based multimetallic nanorings as advanced catalysts.

Here we demonstrate a simple and general approach for the synthesis of Pd@PtM(*M* = Rh, Ni, Pd, Cu) core-shell nanorings in benzaldehyde (BAL) with Pd nanoplates as the templates. The synthesis of the Pd@PtM nanorings with a hollow interior involves in selective epitaxial growth of PtM on the periphery of the as-preformed Pd nanoplates in combination with oxidative etching of Pd in the interior. Compared to the Pd@PtRh nanoplates and commercial Pt/C, the Pd@PtRh nanorings exhibited the substantially enhanced activity for EOR.

## Experimental Section

### Chemicals

Palladium(II) acetylacetonate [Pd(acac)_2_, 99%], platinum(II) acetylacetonate [Pt(acac)_2_, 97%], nickel(II) acetylacetonate [Ni(acac)_2_, 95%], rhodium(III) acetylacetonate [Rh(acac)_3_, 97%], copper(II) acetylacetonate [Cu(acac)_2_, 99.9%], poly(vinylpyrrolidone) (PVP, MW≈29000), oxalic acid (OA), hexadecyltrimethylammonium bromide (CTAB), oleylamine (OAm), and tungsten hexacarbonyl [W(CO)_6_] were all purchased from Sigma Aldrich. Commercial Pt black and Pt/C (20 wt%) were purchased from Alfa Aesar. Benzaldehyde (BAL), benzyl alcohol (BA), dimethylformamide (DMF), ethanol, acetone, chloroform, and toluene were purchased from Sinopharm Chemical Reagent. All syntheses were carried out in a glass flask (25 mL, Shuniu) or home-made 15 mL Teflon-lined stainless-steel autoclave. Deionized water (18.2 MΩ/cm) was used in all experiments. All the chemicals were used as received without further purification.

### Synthesis of 18 nm Pd Nanoplates

Pd nanoplates with average edge length of ~18 nm were synthesized by using a modified protocol based on our previous report (Li et al., 2015b). Typically, 18 mg of Pd(acac)_2_, 30 mg of PVP, 60 mg of CTAB, and 30 mg of OA were dissolved in 10 mL of DMF and the solution was stirred for 1 h at room temperature. Under protection of argon atmosphere, transfer the dissolved red solution to a 25 ml single-necked flask and then add 100 mg of W(CO)_6_. The sealed flask was heated at 60°C under magnet stirring for 3 h and then cooled to room temperature. After then, the solution was stored in argon for future use.

### Synthesis of Pd@PtM (*M* = Rh, Ni, Cu, Pd) Nanorings

For the synthesis of Pd@PtRh nanorings, 300 mg of PVP, 0.015 mmol of Pt(acac)_2_ and 0.005 mmol of Rh(acac)_3_ were dissolved in 10 mL of BAL. Four mL of the solution containing Pd nanoplates was separated by centrifugation with acetone for two times, and re-dispersed in the above-mentioned solution. After that, the resulting mixture was stirred for 1 h and then transferred into a 15 mL Teflon-lined stainless steel autoclave. The autoclave was heated at 200°C for 12 h and then cooled at room temperature. The product was washed and centrifuged with ethanol, repeated three times. Pd@PtNi, Pd@PtCu, and Pd@PtPd nanorings were also synthesized by varying the types and the amount of the metal precursors with other conditions remaining identical one.

### Synthesis of Pd@PtRh Nanoplates

Pd@PtRh nanoplates were synthesized by using a protocol based on our previous report (Yan et al., 2016). In a typical procedure, 300 mg of PVP, 0.015 mmol of Pt(acac)_2_, and 0.005 mmol of Rh(acac)_3_ were dissolved in 10 mL of BA. Four mL of the solution containing Pd nanoplates was separated by centrifugation with acetone, and re-dispersed in the above-mentioned solution. After that, the resulting mixture was stirred for 1h and then transferred into a 15 mL Teflon-lined stainless steel autoclave. The autoclave was heated at 200°C for 12 h and then cooled at room temperature. The product was washed and centrifuged with ethanol, repeated three times.

### Synthesis of Pd@PtRh/C Catalysts

In a standard procedure, the resulting products were phase-transferred into hydrophobic solvent first. The resulting Pd@PtRh nanorings/nanoplates were precipitated by centrifugation with ethanol, and then dispersed in a mixture of ethanol, toluene, and OAm with a volume ratio of 1:1:6. The mixture was heated at 80°C for 12 h in the flow of Ar. The colloidal products were collected by centrifugation, washed with chloroform/ethanol mixture for two times and then was re-dispersed in chloroform. The specific metal content was measured by inductively coupled plasma atomic emission spectroscopy (ICP-AES). Then carbon black (XC-72R) was dispersed in chloroform and sonicated for 30 min. The Pd@PtRh nanorings/nanoplates in chloroform was added to this dispersion. This mixture was further sonicated for 10 min and stirred for 24 h. Then, the resultant was collected by centrifugation and re-dispersed in tert-butylamine. Afterwards, the mixture was stirred for 3 days, and then centrifuged and washed three times with methanol. The resulting catalysts were re-dispersed in deionized water and dried by freeze-drying. The metal loading of the catalysts were determined by thermogravimetric analysis.

### Characterizations

The crystal structure of the Pt-based samples was characterized by X-ray powder diffraction (XRD) using a Rigaku D/max-ga X-ray diffractometer with graphite monochromatized Cu Kα radiation (λ = 1.54178 Å). Transmission electron microscopy (TEM) images of such samples were taken using a HITACHI HT-7700 microscope operated at 100 kV. High-resolution transmission electron microscopy (HRTEM) was performed using a FEI Tecnai F30 G2 microscope operated at 300 kV. High-angle annular dark-field scanning TEM (HAADF-STEM) and Energy dispersive X-ray (EDX) mapping analyses were taken on a FEI Titan ChemiSTEM equipped with a probe-corrector and a Super-X EDX detector system and operated at 200 kV. The percentages of the elements in the samples were determined using inductively coupled plasma atomic emission spectrometry (ICP-AES, IRIS Intrepid II XSP, TJA Co., USA). Gas chromatography mass spectrometer (GC-MS) measurements were performed on a GC-MS 7890A-5975C (Agilent) with molecular ion selective monitoring. All of these samples were diluted with acetone in fixed ratio before the GC-MS measurement.

### Electrochemical Measurements

A three-electrode cell was used to take the electrochemical measurement with a CHI760E electrochemical analyzer (CH Instrument, Shanghai). The working electrode was a glassy-carbon rotating disk electrode (GCE) (diameter: 5 mm and area: 0.196 cm^2^) from Pine Instruments. A platinum wire with the length of 5 cm and a reversible hydrogen electrode (RHE) were used as the counter and reference electrodes, respectively. To prepare the working electrode, water dispersions with 10 μg of pure Pd@PtRh nanorings or Pd@PtRh nanoplates catalysts were dropped on the GCE. After the dispersion was dried by IR-lamp, 3 μL of Nafion solution (0.05%) was deposited on the working electrode. To prepare the inks of the reference catalysts, 5 mg of commercial Pt/C (Alfa Aesar, 20 wt%) were dispersed in a 5 mL of mixed solution containing DI water, isopropanol, 5% Nafion in the volume ratio of 8:2:0.05. After that, the ink was dropped to the GCE and dried in flowing argon. 10 μg metal of all samples was loaded on the GCE. Before the test, the catalysts were electrochemically cleaned by a cyclic voltammogram (CV) process in 0.1 M HClO_4_ solution for 40 cycles. The electrochemically active surface areas (ECSAs) were determined by integrating the hydrogen adsorption charge on CVs. The ECSAs were tested in an argon saturated 0.1 M HClO_4_ solution with a scan rate of 50 mV/s. Ethanol oxidation reaction (EOR) was conducted in a mixture solution containing 0.5 M H_2_SO_4_ and 0.5 M EtOH. The applied potential range for EOR was 0.3–1.1 V (vs. RHE) at a sweep rate of 50 mV/s. The chronoamperometry (I-t) curves were measured at 0.9 V (vs. RHE) for 1000 s.

## Results and Discussion

### Characterization of Pd@PtM (*M* = Rh, Ni, Pd, Cu) Nanorings

**Figure 1:**
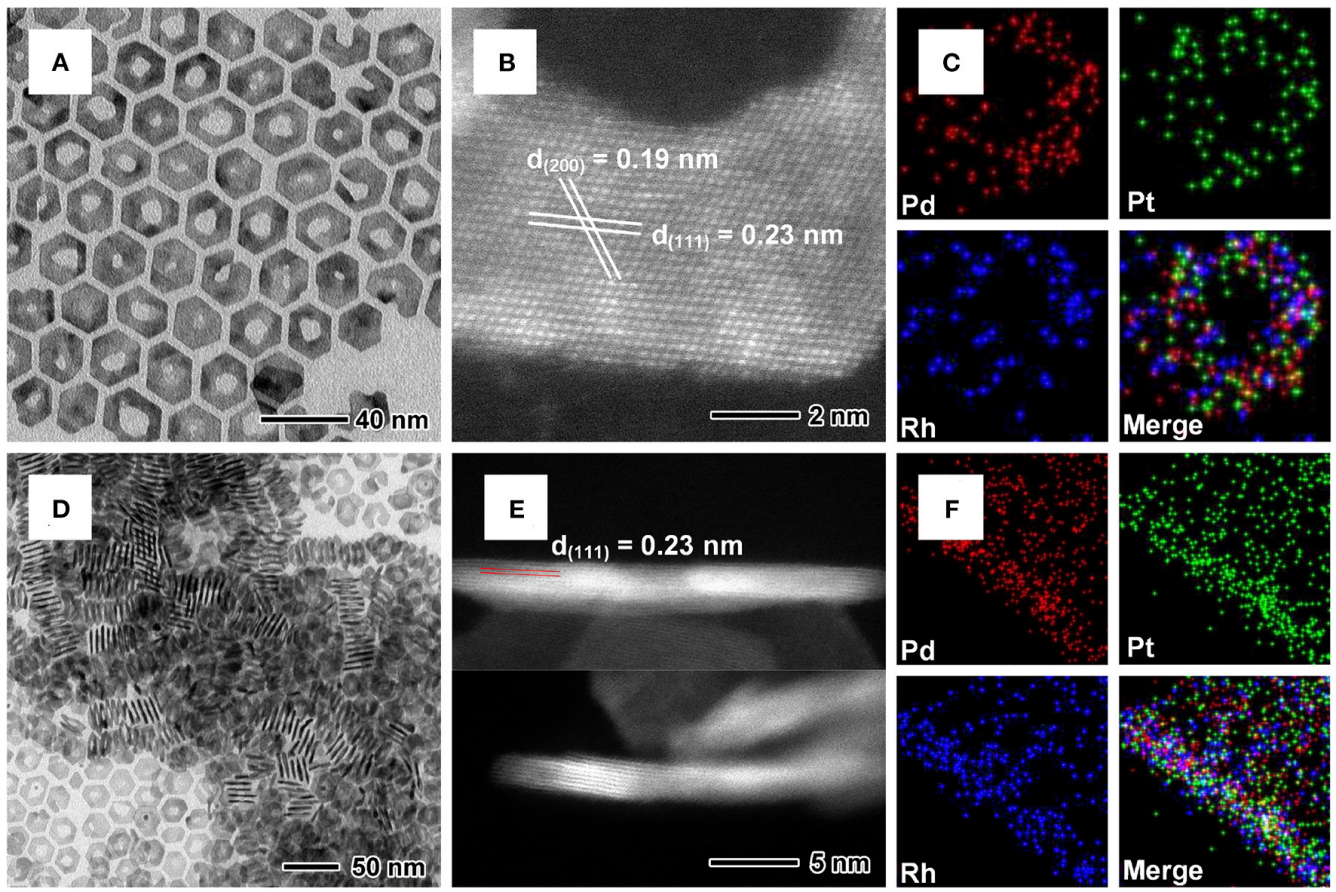
**(A)** TEM, **(B)** atomic-resolution HAADF-STEM, **(C)** EDX mapping images of the planar Pd@PtRh nanorings. **(D)** TEM, **(E)** HAADF-STEM, **(F)** EDX mapping images of the vertically upstanding Pd@PtRh nanorings.

Pd@PtM multimetallic nanorings were synthesized by a simple solvothermal approach in BAL containing the metal precursors and PVP with the Pd nanoplates as the templates. The Pd nanoplates were synthesized by using a similar protocol according to our previous report (Li et al., 2015b). The sizes of the nanoplates were calculated from the TEM image (Supplementary Figure 1), showing the average edge length of ~18.4 nm and thickness of ~1.1 nm. Morphological, structural and compositional characterizations of Pd@PtRh multimetallic nanorings were shown in Figure 1. For better observation, the nanorings were transfer to oil phase. TEM image in Figure 1A shows that most of Pd@PtRh nanorings have a hexagonal shape with an irregular inner hole and present a good morphology uniformity. The HAADF-STEM image (Figure 1B) of an individual planar Pd@PtRh nanoring shows a lattice spacing of 0.23 and 0.19 nm, which can be indexed to {111} and {200} planes of Pd or Pt. The EDX mapping images (Figures 1C,F) of a planar and vertically upstanding Pd@PtRh nanoring show that Pd, Rh, and Pt elements were distribution uniformly, indicating the formation of Pd@PtRh multimetalli c nanorings. The typical HAADF-STEM image (upside of Figure 1E) of a vertically upstanding Pd@PtRh nanoring indicates the continuous lattice fringes from the Pd core to PtRh shell. The inner lattice spacing of 0.23 nm is indexed to {111} planes of Pd, which indicates the epitaxial growth of the PtRh shells on {111} planes of the Pd nanoplates. Moreover, the HAADF-STEM image in Figure 1E upside shows the contrast of both sides is obviously brighter than that of the middle part, which is caused by the central hole of the nanoring structure. The HAADF-STEM image (downside of Figure 1E) shows a clear contrast difference between the Pd core (~5 atomic layers) and PtRh shell (~1–2 atomic layers), further confirming the core-shell structure. Since the thickness of the PtRh alloyed shells is too thin, it is difficult to accurately differentiate the characteristics of the core-shell structure in the EDX mapping image of a Pd@PtRh nanoring in the cross-section view (Figure 1F) and XRD pattern (Supplementary Figure 2).

**Figure 2:**
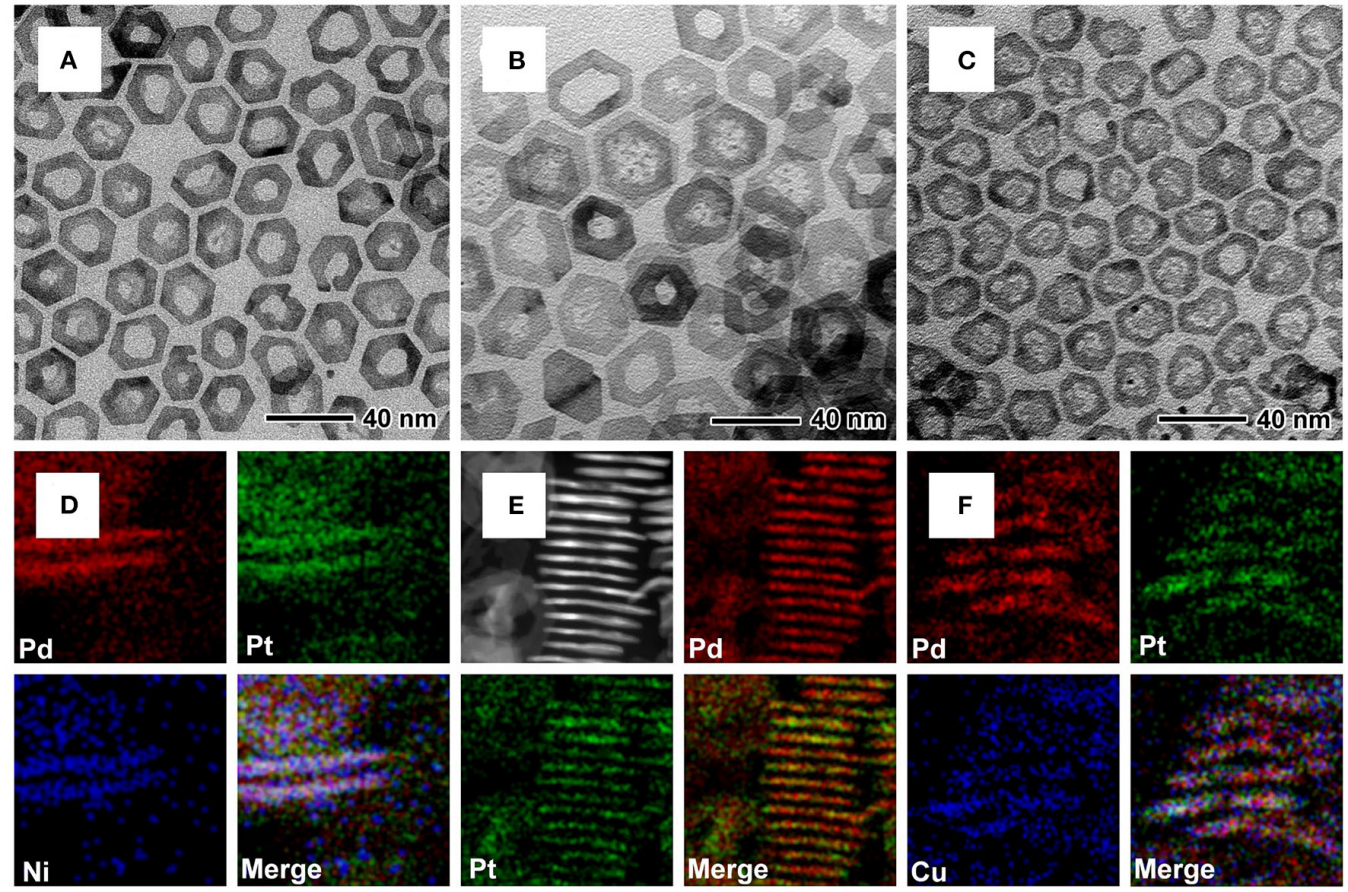
TEM and HAADF-STEM-EDX mapping images for **(A,D)** Pd@PtNi nanorings, **(B,E)** Pd@PtPd nanorings, and **(C,F)** Pd@PtCu nanorings, respectively.

Other multimetallic 2D nanorings such as Pd@PtM (*M* = Ni, Pd, Cu) can also be synthesized by such approach, only varying the type of the metal salt precursors in the synthesis. TEM, HAADF-STEM, and EDX mapping images of vertically standing and planar nanorings were shown in Figure 2 and Supplementary Figure 3, confirmed the epitaxial growth of PtM (*M* = Ni, Pd, Cu) alloy shells on the periphery of the Pd nanoplates with a core-shell structure. However, the TEM images of Pd@PtPd and Pd@PtCu nanorings (Figures 2B,C) show that the Pd in the central part has not been etched completely, leaving some small fragments of Pd. For the Pd@PtPd nanorings, the Pd atoms can be epitaxially deposited on the entire Pd nanoplates due to the homogeneous growth, leading to the increase in the thickness of the Pd nanoplates. For Pd@PtCu nanorings, the introduction of Cu^2+^ ions often induces underpotential deposition of Cu and subsequent galvanic replacement between Cu and Pt^2+^ ions, resulting in the formation of alloys in the central of the Pd nanoplates (Jiang et al., 2015). These two cases lead to the difficulty in complete removal of the Pd nanoplates in the interior. XRD patterns in Supplementary Figure 2 show that all of the diffraction peaks of these nanorings were corresponding to Pd or Pt with an *fcc* structure. ICP-AES data (Supplementary Table 1) show that the atomic ratios of Pt/Rh, Pt/Ni, and Pt/Cu in the multimetallic nanorings are less than the feeding molar ratios of the metal precursors due to the different reduction potential between Pt^2+^ ions and Ni^2+^, Cu^2+^, and Rh^3+^ ions. In summary, these results confirm the universality of the presented approach about using Pd nanoplates as the templates to synthesize Pt-based multimetallic nanorings.

### Formation Mechanism of Pd@PtRh Nanorings

**Figure 3:**
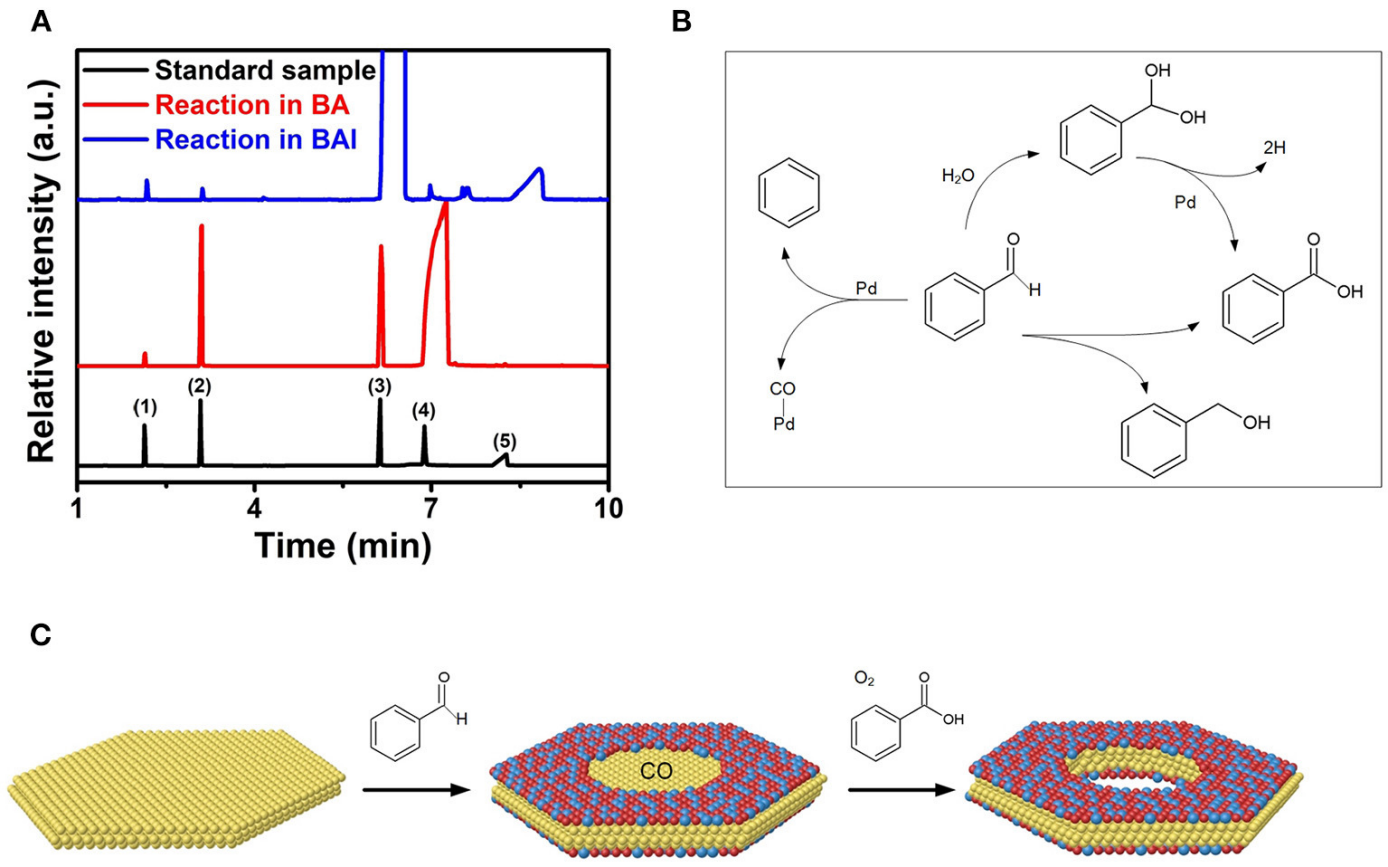
**(A)** GC-MS spectra of three residual solutions including the reaction solution of Pd@PtRh nanorings, nanoplates, and the standard sample containing (1) benzene, (2) toluene, (3) BAl, (4) BA, and (5) benzoic acid. **(B)** Possible reaction pathways for BAL under the solvothermal process in the presence of the Pd nanoplates. **(C)** Scheme for the epitaxial growth and *in-situ* etching of Pd@PtM (M = Rh, Ni, Cu, Pd) nanorings realized by interface catalytic reactions.

Our previous study showed that Pd nanoplates can promote the decomposition of BA into BAL and CO by dehydrogenation in combination with decarboxylation reaction (Yan et al., 2016). The *in-situ* generated CO facilitated the co-reduction and epitaxial growth of Pt-based alloys on Pd nanoplates by remarkably reducing their surface energy through strongly selective adsorption of CO (Eichler, 2002). Here BAL instead of BA was employed as the solvent for the synthesis of Pt-based multimetallic nanorings. Figure 3A shows GC-MS data of residual solutions in the synthesis of the Pd@PtRh nanorings and nanoplates, respectively. It is indicated that much more benzene and benzoic acid were detected in the synthesis of the Pd@PtRh nanorings rather than the corresponding nanoplates. From the possible reaction pathways (Figure 3B), benzene was formed through the decarboxylation of BAL catalyzed by Pd, simultaneously leading to the same molar ratio CO. The appearance of BA in the synthesis of the nanorings indicates that benzoic acid was formed not only through the dehydrogenation process activated by residual water, but also direct decomposition of BAL (Keresszegi et al., 2005; Enache et al., 2006). The *in-situ* generated CO and benzoic acid were critical for the formation of the Pt-based core-shell nanorings.

As well-known, the growth of Pt or Pt-based alloy on a metal (e.g., Pd) thermodynamically adopts an island growth mode instead of epitaxial growth mode because of the restriction from the high surface energy of Pt. Many previous reports confirmed that CO can strongly adsorb on the {111} plane of Pt and Pt-based alloys, leading to the decrease in the surface energy (Ertl et al., 1977; Pick, 1996). This demonstration was supported by the successful epitaxial growth of Pt and Pt-based alloy on Pd nanoplates realized by *in-situ* generated CO through interfacial catalytic reactions (Yan et al., 2016). In this work, more CO was generated through interfacial catalytic reactions by replacing BA with BAL. Such *in-situ* generated CO restricts the deposition of Pt-based alloys on the entire Pd nanoplates due to obstacle effect (Huang et al., 2010; Li et al., 2015a). As such, the epitaxial growth of Pt-based alloys only takes place on the periphery of the Pd nanoplates. This demonstration was supported by the control experiments as follows. Supplementary Figure 4A shows TEM image of the deposition of PtRh on Pd nanoplates prepared in BA by direct inflowing CO at a rate of 50 mL/min into the solution. As observed, PtRh only grows on the periphery of the Pd nanoplates, confirming the obstacle effect from sufficient CO. Simultaneously, the synthesis of Pd@PtRh nanorings was conducted in air atmosphere. The oxygen in air atmosphere in combination with H^+^ ions arising from benzoic acid is a well-known oxidative etching reactant (Yan et al., 2018). The Pd in the interior of the nanoplates without the PtRh shells was removed by H^+^/O_2_ pairs through oxidative etching. When this synthesis was carried out in Ar atmosphere, the Pd in the central of the nanoplates was almost remained, as shown in Supplementary Figure 4B. This result indicates the important role of O_2_ and benzoic acid in the formation of the nanorings. Figure 3C summarizes the formation process of Pd@PtM (*M* = Rh, Ni, Pd, Cu) nanorings. First, a large amount of CO was produced by decarboxylation of benzaldehyde accompanied by the formation of benzoic acid through dehydrogenation or direct decomposition of BAL. Second, the *in-situ* generated CO facilitated not only the co-reduction of the metal precursors, but also the epitaxial growth of PtM alloy on the periphery of the Pd nanoplates. Finally, the Pd in the interior of the nanoplates was removed by oxiditive etching with H^+^/O_2_ pairs, leading to the formation of the nanorings. Compared to the synthesis of the Pd@PtM nanoplates, the use of BAL and existence of oxygen in the solution are critical to the formation of the Pd@PtM nanorings.

### Catalytic Property of Pd@PtRh in Ethanol Oxidation Reaction

**Figure 4:**
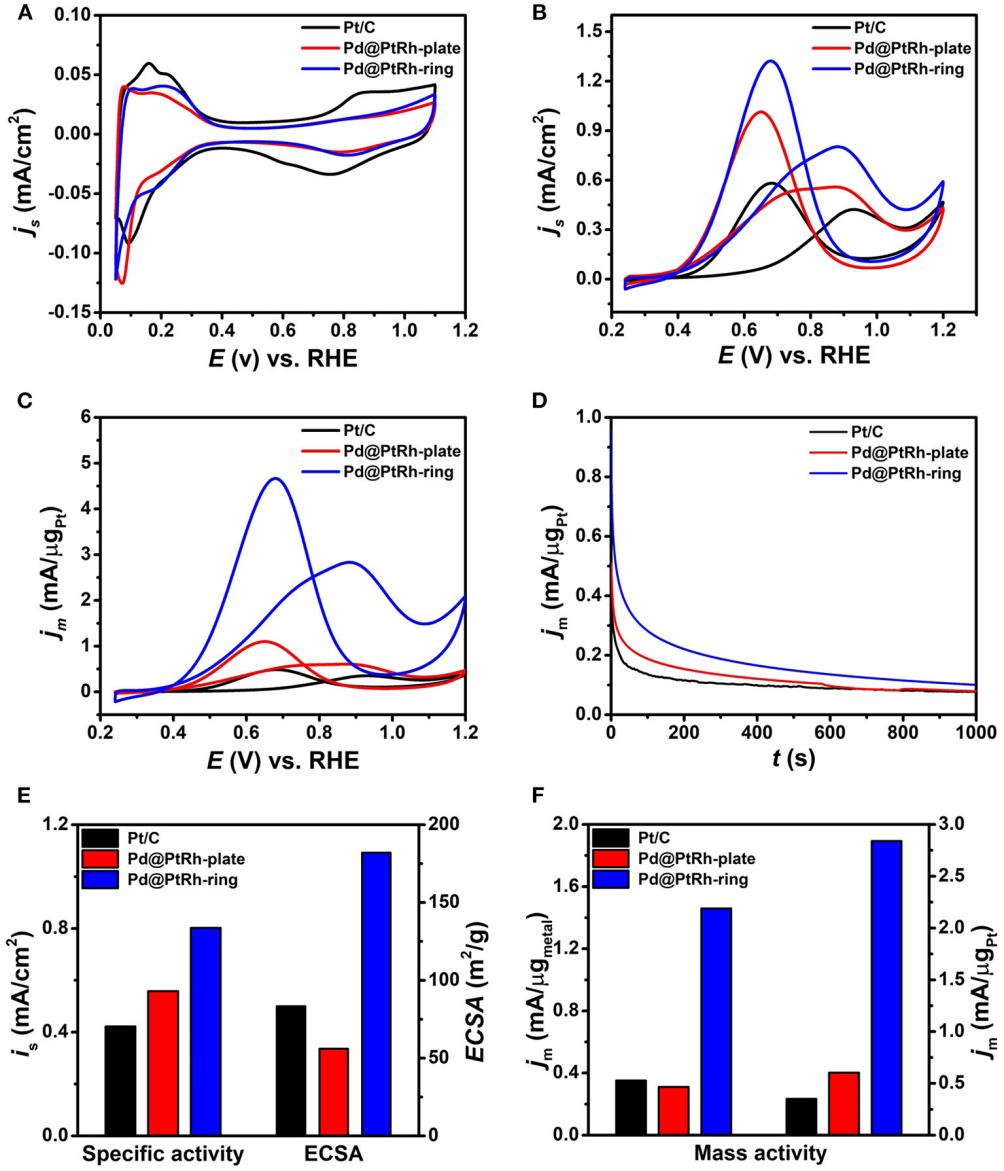
Electrochemical characterization of carbon supported Pd@PtRh nanorings, Pd@PtRh nanoplates, and Pt/C catalysts: **(A)** CV curves in 0.1 M HClO_4_ solution at a scan rate of 50 mV/s; **(B,C)** CV curves normalized with ECSAs and Pt mass, respectively, in a mixed solution containing 0.5 M H_2_SO_4_ and 0.5 M EtOH at a scan rate of 50 mV/s; **(D)** i-t curves at 0.9 V (vs. RHE) for 1000 s; **(E)** specific activities and ECSAs; **(F)** mass activities normalized with metals and Pt mass, respectively.

The Pd@PtRh nanorings were selected as catalysts for EOR in acid media with the Pd@PtRh nanoplates and commercial Pt/C as references. TEM images in Supplementary Figure 5 show the well dispersion of Pd@PtRh nanoplates and nanorings on carbon supports. The electrochemically active surface areas (ECSAs) of these three catalysts were calculated from the adsorption and desorption charge of a single layer of H_UPD_ in CV curves performed in an Ar-saturated HClO_4_ solution (Figure 4A). As observed from Supplementary Table 2 and Figure 4E, the Pd@PtRh nanoplates and Pt/C had a closed ECSAs, while the Pd@PtRh nanorings exhibited much larger ECSAs than others. This result can be attributed to the unique ring-like structure with both exterior and interior catalytic surfaces. In addition, two-dimensional nanostructures (e.g., nanoplates and nanorings) thermodynamically tend to be assembled in a face-to-face way in an aim to reduce the surface energy, which was supported by TEM images in Supplementary Figure 6. This self-assembly can reduce their ECSAs to some content. However, the hollow structure in the nanorings can dramatically weaken the loss of ECSAs, leading to the much larger ECSAs of the nanorings than nanoplates. Figures 4B,C compare CV curves of these three catalysts in a mixed solution containing 0.5 M H_2_SO_4_ and 0.5 M EtOH at a scan rate of 50 mV/s for EOR normalized by ECSA and Pt mass, respectively. The corresponding specific and mass activities are summarized in Figures 4E,F and Supplementary Table 2. Compared to the commercial Pt/C, both Pd@PtRh nanoplates and nanorings showed the substantially enhanced catalytic activities for EOR. Specifically, the Pd@PtRh nanorings show the highest specific (0.802 mA/cm^2^) and mass (3.07A/mg_Pt_) activities, which are ~1.4- and ~4.9-times higher than those of Pd@PtRh nanoplates and ~1.9- and ~8.7-times higher than those of commercial Pt/C, respectively. The enhanced EOR activities of Pd@PtRh nanoplates and nanorings can be understood by well-known bifunctional mechanism (Yajima et al., 2004; Zhu et al., 2016). The introduction of oxophilic Rh and Pd can dissociate water to form the oxygenated species such as OH_ads_, thereby improving the catalytic activity through alleviating CO poisoning on Pt active sites. In addition, the interior part of the nanorings contains a large number of active sites, unsaturated bonds and high-index facets, which are mainly responsible for the difference in specific activities between Pd@PtRh nanorings and nanoplates. This demonstration was supported by our previous report in which such edge sites have proved to be highly active for catalytic reaction (Yan et al., 2018). The much larger ECSAs of the Pd@PtRh nanorings rather than the nanoplates eventually leads to the huge difference in mass activity.

The stability of these three catalysts toward EOR was tested by chronoamperometric experiments performed at 0.9 V for 1000 s. Current-time (I-t) curves (Figure 4D) shows that the Pd@PtRh nanorings exhibit the higher steady current density than Pd@PtRh nanoplates and Pt/C, respectively. This result indicates the superior durability of the nanorings. In addition, the Pd@PtRh nanoplates have more performance degradation than commercial Pt/C. From TEM images of the Pd@PtRh nanoplates and nanorings after the electrochemical measurements (Supplementary Figure 7), some Pd@PtRh nanoplates were turned into fragments by etching, while most of Pd@PtRh nanorings retained their shapes. This result indicates that the structural damage of multimetallic nanoplates with atomic thickness under electrochemical test conditions is the main reason for their performance degradation.

## Conclusion

In summary, we have designed a general approach for the synthesis for Pt-based multimetallic nanorings with tunable compositions through epitaxial growth in combination with oxidation etching using Pd nanoplates as the templates. The key to such ring-like structure is *in situ* production of CO and benzoic acid from BAL through interface catalytic reactions catalyzed by Pd nanoplates. Sufficient CO promotes the epitaxial growth of Pt-based alloys on the periphery of Pd nanoplates by decreasing the surface energy through strong adsorption. Simultaneously, the Pd in the interior of the Pd nanoplates was removed by oxidation etching arising from benzoic acid and oxygen, eventually leading to the formation of the nanorings. The Pd@PtRh multimetallic nanorings show substantially enhanced catalytic performance in terms of activity and durability toward EOR compared to the benchmark catalysts such as Pd@PtRh nanoplates and Pt/C due to the unique hollow structure. This work not only provides a general approach for the synthesis of the nanorings made of multi-metals, but also o?ers a fresh impetus for the rational design of advanced catalysts with rich active sites.

## Data Availability Statement

The original contributions presented in the study are included in the article/Supplementary Material, further inquiries can be directed to the corresponding author.

## Author Contributions

All authors listed have made a substantial, direct and intellectual contribution to the work, and approved it for publication.

## Conflict of Interest

The authors declare that the research was conducted in the absence of any commercial or financial relationships that could be construed as a potential conflict of interest.

## References

[B1] Antolini E. (2007). Catalysts for direct ethanol fuel cells. J. Power Sources 170:1. 10.1016/j.jpowsour.2007.04.00925338505

[B2] Brouzgou A. Podias A. Tsiakaras P. (2012). PEMFCs and AEMFCs directly fed with ethanol: a current status comparative review. J. Appl. Electrochem. 43, 119–136. 10.1007/s10800-012-0513-2

[B3] Chee S. Tan S. Baraissov Z. Bosman M. Mirsaidov U. (2017). Direct observation of the nanoscale kirkendall effect during galvanic replacement reactions. Nat. Commun. 8:1224. 10.1038/s41467-017-01175-2 29089478 PMC5663914

[B4] Chen Y. Fan Z. Zhang Z. Niu W. Li C. Yang N.. (2018). Two-dimensional metal nanomaterials: synthesis, properties, and applications. Chem. Rev. 118, 6409–6455. 10.1021/acs.chemrev.7b00727 29927583

[B5] De Souza J. P. I. Queiroz S. L. Bergamaski K. Gonzalez E. R. Nart F. C. (2002). Electro-oxidation of ethanol on Pt, Rh, and PtRh electrodes. A study using DEMS and *in-situ* FTIR techniques. J. Phys. Chem. B 106:9825. 10.1021/jp014645c

[B6] Eichler A. (2002). CO oxidation on transition metal surfaces: reaction rates from first principles. Surf. Sci. 498, 314–320. 10.1016/S0039-6028(01)01805-2

[B7] Enache D. Edwards J. Landon P. Solsona-Espriu B. Carley A. Herzing A.. (2006). Solvent-free oxidation of primary alcohols to aldehydes using Au-Pd/TiO_2_ catalysts. Science 311, 362–365. 10.1126/science.1120560 16424335

[B8] Ertl G. Neumann M. Streit K. (1977). Chemisorption of CO on the Pt (111) Surface. Surf. Sci. 64, 393–410. 10.1016/0039-6028(77)90052-8

[B9] Fan N. Yang Y. Wang W. Zhang L. Chen W. Zou C.. (2012). Selective etching induces selective growth and controlled formation of various platinum nanostructures by modifying seed surface free energy. ACS Nano 6, 4072–4082. 10.1021/nn3004668 22506898

[B10] Fan Z. Huang X. Tan C. Zhang H. (2015). Thin metal nanostructures: synthesis, properties, and applications. Chem. Sci. 6, 95–111. 10.1039/C4SC02571G 28553459 PMC5424468

[B11] Farsadrooh M. Torrero J. Pascual L. Peña M. Retuerto M. Rojas S. (2018). Two-dimensional Pd-nanosheets as efficient electrocatalysts for ethanol electrooxidation. Evidences of the C-C scission at low potentials. Appl. Catal. B Environ. 237, 866–875. 10.1016/j.apcatb.2018.06.051

[B12] Huang J. Yan Y. Li X. Qiao X. Wu X. Li J.. (2020). Unexpected kirkendall effect in twinned icosahedral nanocrystals driven by strain tradient. Nano Res. 13, 2641–2649. 10.1007/s12274-020-2903-9

[B13] Huang X. Tang S. Mu X. Dai Y. Chen G. Zhou Z.. (2010). Freestanding palladium nanosheets with plasmonic and catalytic properties. Nat. Nanotech. 6, 28–32. 10.1038/nnano.2010.235 21131956

[B14] Iwasita T. Pastor E. (1994). A dems and FTIR spectroscopic investigation of adsorbed ethanol on polycrystalline platinum. Electrochim. Acta 39:531. 10.1016/0013-4686(94)80097-9

[B15] Jiang Y. Bian T. Lin F. Zhang H. Jin C. Li Z.. (2015). Revealing the elemental-specific growth dynamics of Pt–Cu multipods by scanning transmission electron microscopy and chemical mapping. J. Mater. Chem. A 3, 21284–21289. 10.1039/C5TA05721C

[B16] Keresszegi C. Ferri D. Mallat T. Baiker A. (2005). Unraveling the surface reactions during liquid-phase oxidation of benzyl alcohol on Pd/Al_2_O_3_: an *in-situ* ATR-IR study. J. Phys. Chem. B 109, 958–967. 10.1021/jp0459864 16866465

[B17] Kua J. Goddard W. A. (1999). Oxidation of methanol on 2nd and 3rd row group VIII transition metals (Pt, Ir, Os, Pd, Rh, and Ru): application to direct methanol fuel cells. J. Am. Chem. Soc. 121, 10928–10941. 10.1021/ja9844074

[B18] Li C. Tan H. Lin J. Luo X. Wang S. You J.. (2018). Emerging Pt-based electrocatalysts with highly open nanoarchitectures for boosting oxygen reduction reaction. Nano Today 21, 91–105. 10.1016/j.nantod.2018.06.005

[B19] Li Y. Wang W. Xia K. Zhang W. Jiang Y. Zeng Y.. (2015a). Ultrathin two-dimensional Pd-based nanorings as catalysts for hydrogenation with high activity and stability. Small 11, 4745–4752. 10.1002/smll.201500769 26150015

[B20] Li Y. Yan Y. Li Y. Zhang H. Li D. Yang D. (2015b). Size-controlled synthesis of Pd nanosheets for tunable plasmonic properties. CrystEngComm 17, 1833–1838. 10.1039/C4CE02062F

[B21] Liu M. Lu Y. Chen W. (2013). PdAg nanorings supported on graphene nanosheets: highly methanol-tolerant cathode electrocatalyst for alkaline fuel cells. Adv. Funct. Mater. 23, 1289–1296. 10.1002/adfm.201202225

[B22] Pick Š. (1996). Tight-binding model of CO adsorption at PtNi (111) surface. Surf. Sci. 352, 300–304. 10.1016/0039-6028(95)01151-X

[B23] Prieto G. Tüysüz H. Duyckaerts N. Knossalla J. Wang G. Schüth F. (2016). Hollow nano- and microstructures as catalysts. Chem. Rev. 116, 14056–14119. 10.1021/acs.chemrev.6b00374 27712067

[B24] Rossmeisl J. Ferrin P. Tritsaris G. A. Nilekar A. U. Koh S. Bae S. E.. (2012). Bifunctional anode catalysts for direct methanol fuel cells. Energy Environ. Sci. 5, 8335–8342. 10.1039/c2ee21455e

[B25] Sun Y. Mayers B. Xia Y. (2002). Template-engaged replacement reaction: a one-step approach to the large-scale synthesis of metal nanostructures with hollow interiors. Nano Lett. 2, 481–485. 10.1021/nl025531v

[B26] Sun Y. Wiley B. Li Z. Xia Y. (2004). Synthesis and optical properties of nanorattles and multiple-walled nanoshells/nanotubes made of metal alloys. J. Am. Chem. Soc. 126, 9399–9406. 10.1021/ja048789r 15281832

[B27] Wang W. Zhang X. Zhang Y. Chen X. Ye J. Chen J.. (2020). Edge enrichment of ultrathin 2D PdPtCu trimetallic nanostructures effectuates top-ranked ethanol electrooxidation. Nano Lett. 20, 5458–5464. 10.1021/acs.nanolett.0c01908 32492344

[B28] Wang X. Figueroa-Cosme L. Yang X. Luo M. Liu J. Xie Z.. (2016a). Pt-based icosahedral nanocages: using a combination of {111} facets, twin defects, and ultrathin walls to greatly enhance their activity toward oxygen reduction. Nano Lett. 16, 1467–1471. 10.1021/acs.nanolett.5b05140 26760681

[B29] Wang X. Luo M. Huang H. Chi M. Howe J. Xie Z.. (2016b). Facile synthesis of Pt–Pd alloy nanocages and Pt nanorings by templating with Pd nanoplates. ChemNanoMat 2, 1086–1091. 10.1002/cnma.201600238

[B30] Xia X. Wang Y. Ruditskiy A. Xia Y. (2013). Galvanic replacement: a simple and versatile route to hollow nanostructures with tunable and well-controlled properties. Adv. Mater. 25, 6313–6333. 10.1002/adma.201302820 24027074

[B31] Xia Y. Gilroy K. Peng H. Xia X. (2017). Seed-mediated growth of colloidal metal nanocrystals. Angew. Chem. Int. Ed. 56, 60–95. 10.1002/anie.201604731 27966807

[B32] Yajima T. Uchida H. Watanabe M. (2004). *In-situ* ATR-FTIR spectroscopic study of electro-oxidation of methanol and adsorbed CO at Pt–Ru alloy. J. Phys. Chem. B 108, 2654–2659. 10.1021/jp037215q

[B33] Yan Y. Li X. Tang M. Zhong H. Huang J. Bian T.. (2018). Tailoring the edge sites of 2D Pd nanostructures with different fractal dimensions for enhanced electrocatalytic performance. Adv. Sci. 5:1800430. 10.1002/advs.201800430 30128248 PMC6096982

[B34] Yan Y. Shan H. Li G. Xiao F. Jiang Y. Yan Y.. (2016). Epitaxial growth of multimetallic Pd@PtM (M= Ni, Rh, Ru) core–shell nanoplates realized by *in situ*-produced CO from interfacial catalytic reactions. Nano Lett. 16, 7999–8004. 10.1021/acs.nanolett.6b04524 27960487

[B35] Yang X. Roling L. Vara M. Elnabawy A. Zhao M. Hood Z.. (2016). Synthesis and characterization of Pt–Ag alloy nanocages with enhanced activity and durability toward oxygen reduction. Nano Lett. 16, 6644–6649. 10.1021/acs.nanolett.6b03395 27661446

[B36] Zhang L. Doyle-Davis K. Sun X. (2019). Pt-based electrocatalysts with high atom utilization efficiency: from nanostructures to single atoms. Energy Environ. Sci. 12, 492–517. 10.1039/C8EE02939C

[B37] Zhang L. Roling L. Wang X. Vara M. Chi M. Liu J.. (2015). Platinum-based nanocages with subnanometer-thick walls and well-defined, controllable facets. Science 349, 412–416. 10.1126/science.aab0801 26206931

[B38] Zhu C. Shi Q. Fu S. Song J. Xia H. Du D.. (2016). Efficient synthesis of MCu (M = Pd, Pt, and Au) aerogels with accelerated gelation kinetics and their high electrocatalytic activity. Adv. Mater. 28, 8779–8783. 10.1002/adma.201602546 27546519

