# Supplementary material for: Facile Synthesis of Pd@PtM (M = Rh, Ni, Pd, Cu) Multimetallic Nanorings as Efficient Catalysts for Ethanol Oxidation Reaction
Source: Front Chem. 2021 May 19;9:683450. doi: 10.3389/fchem.2021.683450 (PMC8170318; doi:10.3389/fchem.2021.683450)
Supplement: Supplementary file 1 [file Data_Sheet_1.docx]

Supplementary Material


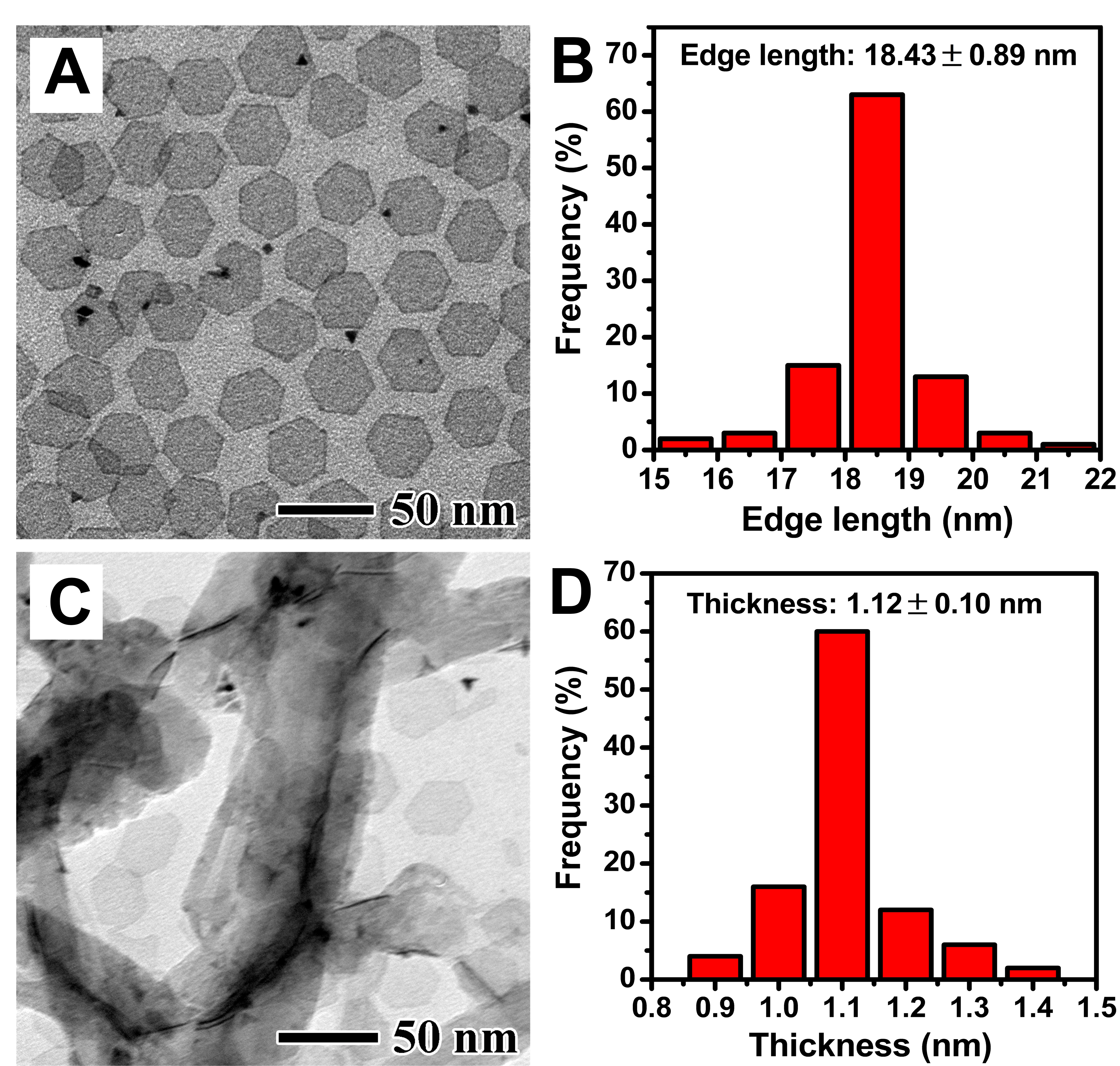


**Figure S1.** TEM images of the Pd nanoplates seeds that (A) lay flat on the TEM grid and (C) attached vertically on the carbon nanotubes. The corresponding (B) edge length and (D) thickness distributions of the Pd seeds.





**Figure S2.** The XRD patterns of the Pd@PtM (M = Rh, Ni, Pd, or Cu) multimetallic nanorings.


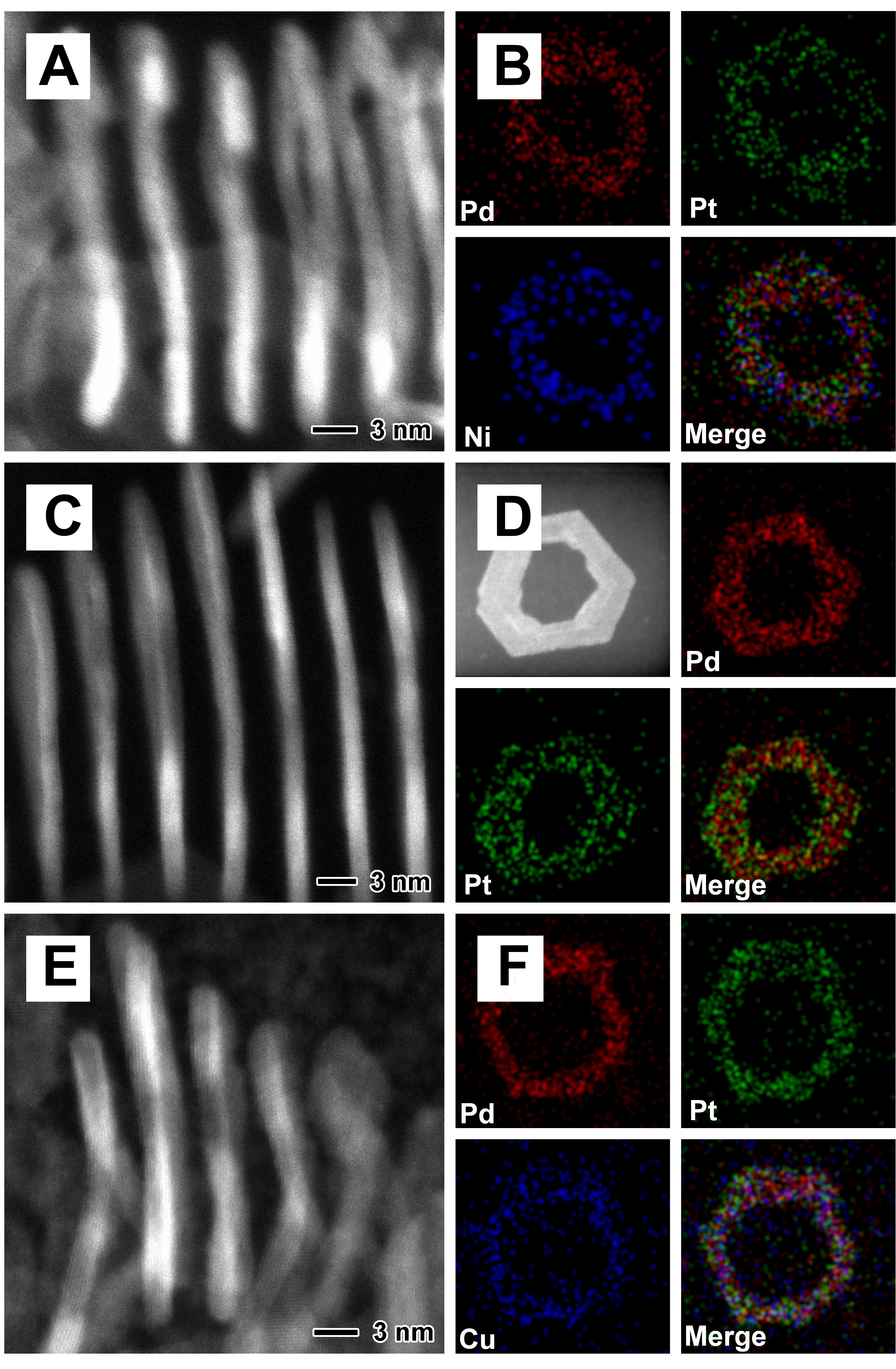


**Figure S3.** HAADF-STEM images of the vertically standing nanorings and EDX mapping images of the planar nanorings for (A, B) Pd@PtNi nanorings, (C, D) Pd@PtPd nanorings, and (E, F) Pd@PtCu nanorings, respectively.


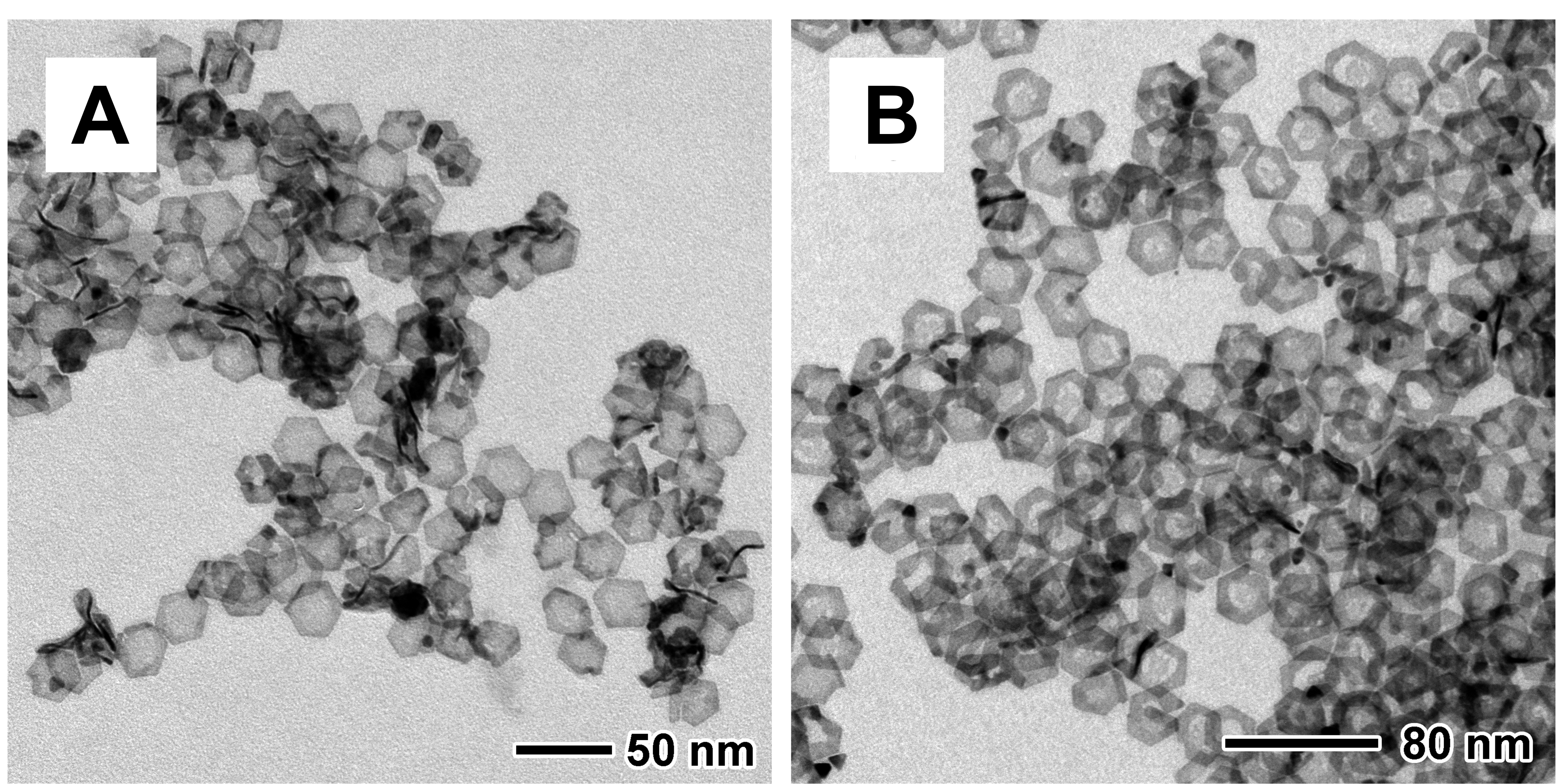


**Figure S4.** TEM images of Pd@PtRh nanoplates prepared in (A) CO atmosphere and BA solution, (B) Ar atmosphere and BAL solution


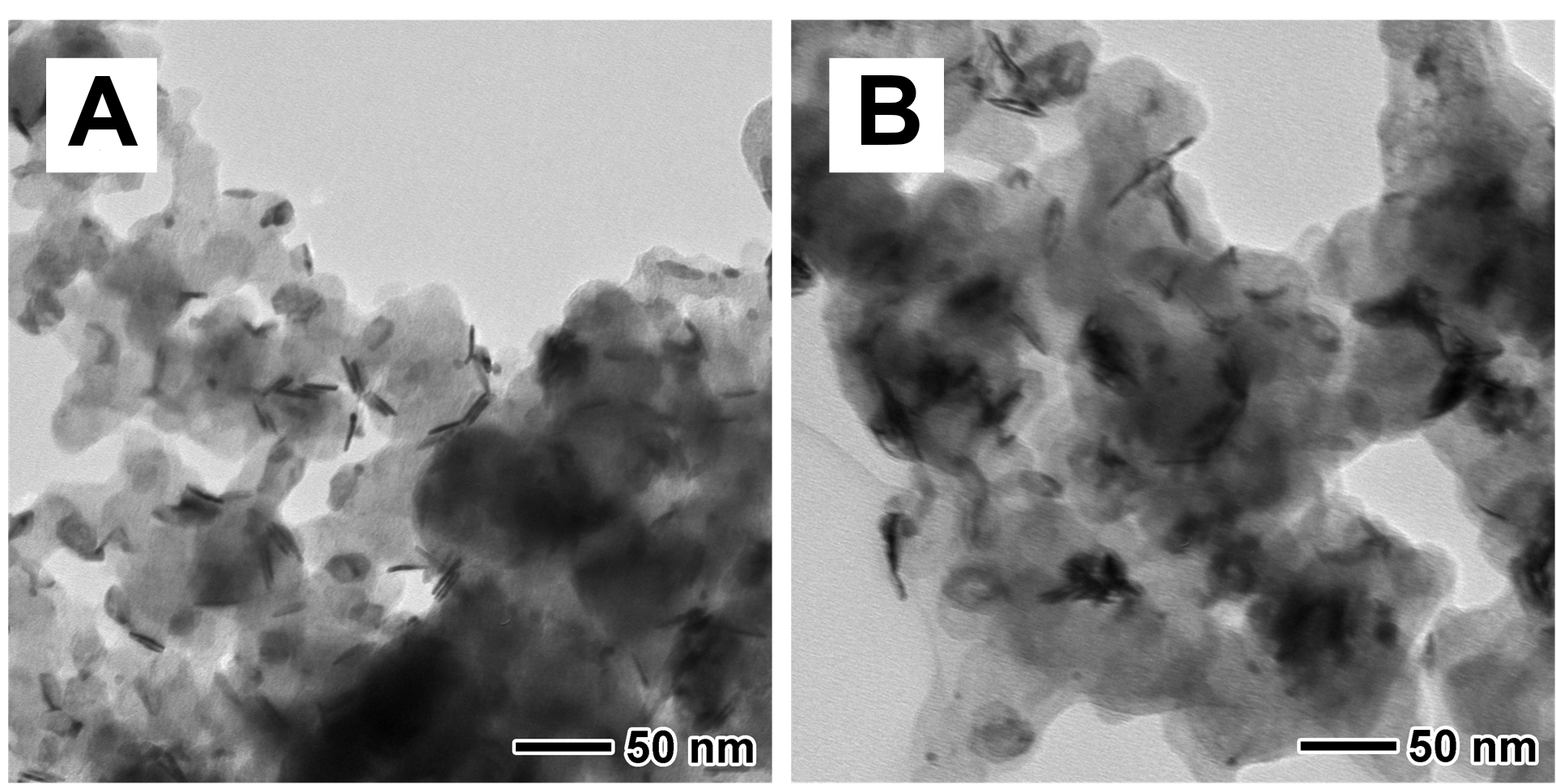


**Figure S5.** TEM images of carbon supported (A) Pd@PtRh nanoplate and (B) Pd@PtRh nanoring catalysts.


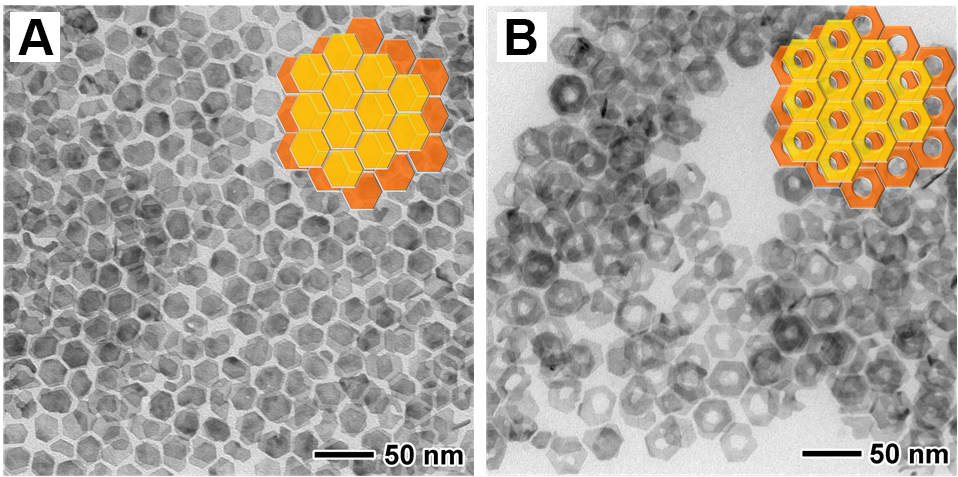


**Figure S6.** TEM images of a few layers of (A) Pd@PtRh nanoplates and (B) nanorings which are both assembled in a face-to-face way. The insets show the corresponding models.


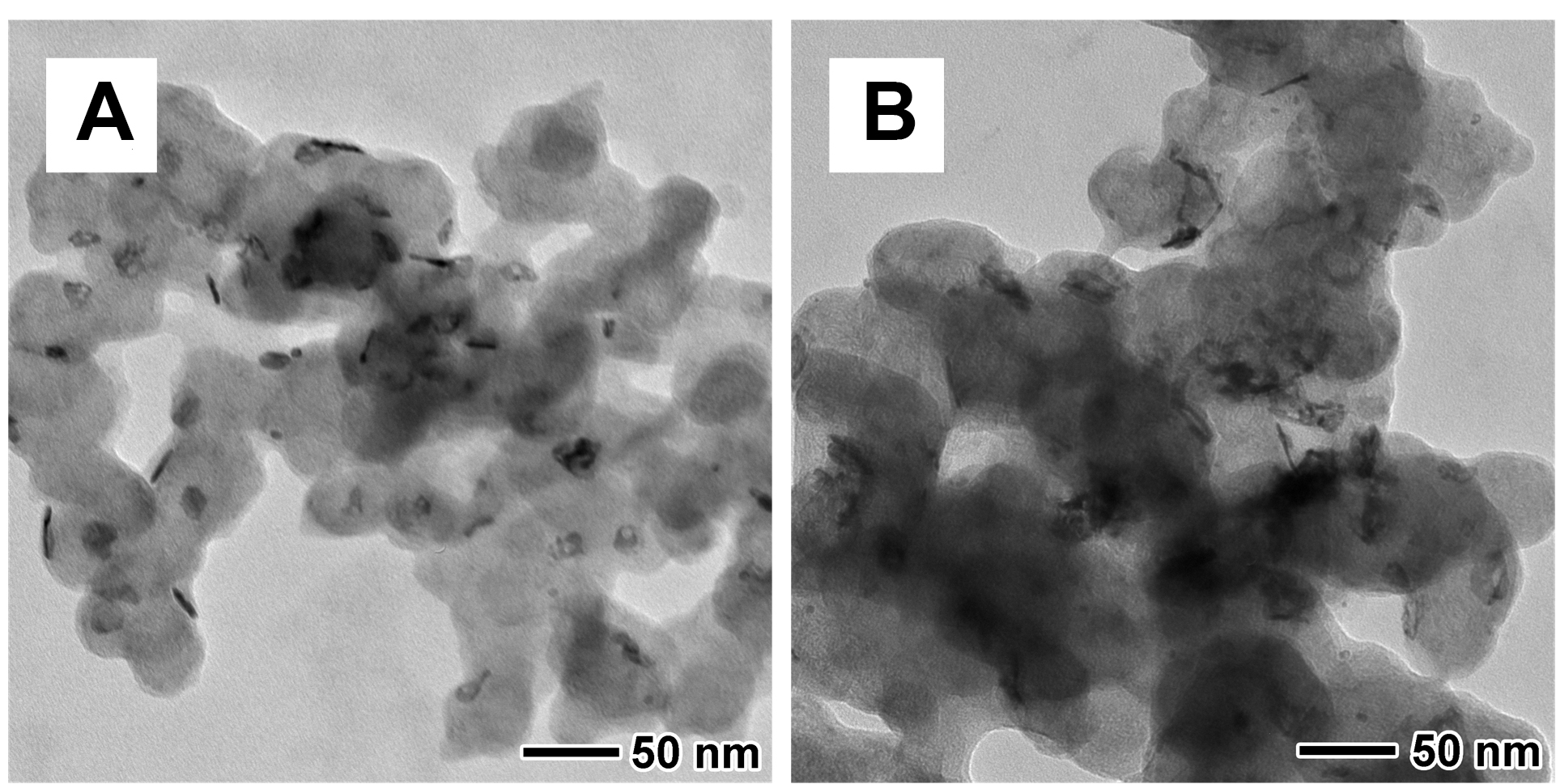


**Figure S7.** Representative TEM images for the (A) Pd@PtRh nanoplates and (B) Pd@PtRh nanorings after electrochemical durability tests.

Table S1. ICP-AES data of the Pd@PtM (M = Rh, Ni, Cu) nanorings for Pt/M ratio.

| Pt : M  (M = Rh, Ni, Cu) | molar ratio of the metal  precursors | atomic ratio of Pt/M |
| --- | --- | --- |
| Pd@PtRh | 75 : 25 | 88.2 : 11.8 |
| Pd@PtNi | 50 : 50 | 72.9 : 27.1 |
| Pd@PtCu | 50 : 50 | 67.7 : 32.3 |

Table S2. ECSAs of Pd@PtRh nanorings, nanoplates and Pt/C calculated from H_UPD_ charge, and the specific and mass activities of these three catalysts for EOR.

| Samples | ECSA (m^2^/g) | *i*_s_ (mA/cm^2^) | *i*_m_ (A/mg_metal_) | *i*_m_ (A/mg_Pt_) |
| --- | --- | --- | --- | --- |
| Pt/C | 83.3 | 0.422 | 0.352 | 0.352 |
| Pd@PtRh nanoplates | 56 | 0.558 | 0.31 | 0.63 |
| Pd@PtRh nanorings | 182 | 0.802 | 1.46 | 3.07 |

Table S3. Comparison of EOR performance of Pd@PtRh nanorings in this work with state of art electrocatalysts in the literatures.

| Samples | Conditon | *i*_s_ (mA/cm^2^) | *i*_m_ (A/mg_Pt_) | Ref. |
| --- | --- | --- | --- | --- |
| Pd@PtRh nanorings | 0.5 M H_2_SO_4_ + 0.5 M EtOH | 0.802 | 3.07 | This work |
| Pt_3_Sn NFs-L/C | 0.1 M HClO_4_ + 0.5 M EtOH | 2.10 | 1.46 | ACS Catal. 2020, 10, 5, 3455–3461 |
| Rh @ Pt_3.5L_ | 0.1 M HClO_4_ + 0.2 M EtOH | 0.81 | 1.18 | Adv. Funct. Mater. 2019, 29, 1806300 |
| Pt–Rh–Ni/C | 0.5 M H_2_SO_4_ + 0.5 M EtOH | / | 0.65 | Electrochimica Acta 2020, 351, 136223 |
| Rh@Pt d-CNCs/C | 0.1 M HClO_4_ +0.2 M EtOH | 2.62 | 0.86 | J. Mater. Chem. A, 2019,7, 17987-17994 |
| Pt-0.5SnO_x_/NCNC | 0.5 M H_2_SO_4_ + 0.5 M EtOH | / | 1.19 | ACS Catal. 2018, 8, 9, 8477–8483 |
| Pt-Mo-Ni NWs | 0.5 M H_2_SO_4_ + 2 M EtOH | 2.57 | 0.87 | Sci. Adv. 2017, 3, e1603068-e1603076 |
